# Antibacterial Chemical Constituent and Antiseptic Herbal Soap from *Salvinia auriculata* Aubl.

**DOI:** 10.1155/2013/480509

**Published:** 2013-12-29

**Authors:** Samia Lima, Gaspar Diaz, Marisa Alves Nogueira Diaz

**Affiliations:** ^1^Department of Biochemistry and Molecular Biology, Federal University of Viçosa, 36570-000 Viçosa, MG, Brazil; ^2^Department of Chemistry, Federal University of Minas Gerais, 31270-901 Belo Horizonte, MG, Brazil

## Abstract

The bioassay-guided isolation of the active extract of *Salvinia auriculata* Aubl. led to the separation of three main compounds, characterized as stigmasterone, stigmasterol, and friedelinol. The pure form of diketosteroid presented a potential antibacterial activity with a minimum inhibitory concentration (MIC) value of 0.01 mg mL^−1^ against *Staphylococcus aureus* isolated from animals with mastitis infections. The active extract also showed a similar result to that previously obtained with pure diketosteroid when tested with the same isolates. The present study's results demonstrate the potential of this plant as an excipient for the production of antibacterial soaps aimed at controlling bovine mastitis infections, especially on small farms.

## 1. Introduction

In recent decades, research on plants with antimicrobial properties has intensified, due mainly to the fact that these plants are considered sources of pharmacologically active compounds that can become new antibiotics after their pure constituents or active extracts have been evaluated [1]. Plants that live in nutrient-rich environments, as well as plants living in environments containing exceptionally high bacterial cell density (i.e., aquatic plants), will be overwhelmed by microbial biofilms if they lack any means of biofilm control [2, 3]. For this reason, aquatic plants have attracted the interest of researchers and have proven to be promising sources of antimicrobial agents [4].

*Salvinia auriculata *Aubl. (Salviniaceae), also known as *orelha de onça*, is a native aquatic plant from South America and is commonly found in freshwater lakes [5]. Currently few studies show the potential of *S. auriculata* in the remediation of water sources contaminated with heavy metals [6], and no studies in the literature report on the antimicrobial agents of their chemical constituents and active extracts.

In a preliminary study performed with this plant, the present study's research group evaluated the antimicrobial potential of extracts obtained from the roots and leaves of this plant against Gram-positive bacteria associated with bovine mastitis. The subsequent results illustrated a strong potential of this plant to combat *Staphylococcus aureus*, the main etiologically causative agent of bovine mastitis [7]. This disease, an inflammatory response found in cows' udders, is the leading infectious disease affecting dairy cattle today [8]. According to the Brazilian Department of Agriculture, while Brazil produces nearly 27 billion gallons/year of milk to be distributed worldwide, the search for the control and treatment of this disease presents a major issue for both political policies and the Brazilian economy [9]. Thus, this work aims to examine the bioassay-guided isolation of compounds responsible for antibacterial activity from active extract and to evaluate the antiseptic potential of an herbal soap produced with *S. auriculata*.

## 2. Materials and Methods

### 2.1. General

Silica gel (70–230 mesh) and glass columns were used for column chromatography. All of the solvents used were of analytical grade. The melting point was determined using a Thermopan apparatus (C. Reichest Optische Wercke A G). ^1^H and ^13^C nuclear magnetic resonance (NMR) spectra were recorded on 300 MHz and 75 MHz NMR spectrometers, respectively (Mercury 300 spectrometer). Tetramethylsilane (TMS) was used as an internal standard. HR-EI-MS spectra were obtained in a microTOFQ II Bruker Daltonics spectrometer. The IR spectra were measured in a Perkin Elmer Paragon 1000 FTIR spectrophotometer, using potassium bromide (1% w/w) scanning from 400 to 4000 cm^−1^.

### 2.2. Plant Material

*S. auriculata *was collected from a pond located in Recanto das Cigarras (20°45′27′′S, 45°51′46′′W), Federal University of Viçosa, Minas Gerais, Brazil, from December 2010 to February 2011. An authenticated voucher (VIC 32.122) was deposited in the university's herbarium. After the material has been exhaustively washed in water, the roots and leaves were separated. The plant parts were dried at 40°C for 24 h in an air circulation oven, and 800 g of roots was extracted using *n*-hexane for 2 days and repeated at least five times. The solvent was concentrated under reduced pressure until it was completely dry and stored at 4°C.

### 2.3. Phytochemical Studies

The crude extract of the roots was dissolved in a minimum amount of hexane and adsorbed on silica gel (70–230 mesh). The extract was subjected to a chromatography column using *n*-hexane as a mobile phase. The eluent polarity was then gradually increased by adding CH_2_Cl_2_, yielding 58 fractions, which subsequently underwent biological assay using the *S. aureus *strain 4127 (identified by Embrapa Dairy Cattle—Milk Microbiology Laboratory) as a reference microorganism. The positive fraction was submitted to a preparative thin layer chromatography (PTLC) eluted with petrol/EtOAc 8 : 2, allowing for the isolation of **1** (6.5 mg), **2** (27.0 mg), and **3** (5.5 mg) compounds.

### 2.4. Bacterial Strains and Culture Media

The bacterial strains used in this study, which were isolated from animals with mastitis infections, were kindly provided by the Embrapa Dairy Cattle—Milk Microbiology Laboratory (Juiz de Fora, Minas Gerais, Brazil). Five *S. aureus *strains (582, 680, 2221, 4006, and 4127) were used to determine the antimicrobial activity of the fractions and isolated compounds. Bacteria were routinely cultured on brain heart infusion (BHI) agar at 37°C for 16 h before conducting the experiments. The cell concentration was adjusted to 10^6^ CFU mL^−1^ with an optical density set at 600 nm. Stock cultures were maintained in BHI agar containing 25% glycerol at −80°C.

### 2.5. Antibacterial Screening Assay

Hole-plate diffusion assay was initially performed to test the antibacterial activity of the fractions obtained from crude extracts of the roots. To accomplish this, the bacteria were cultivated overnight, and a suspension containing 10^6^ CFU mL^−1^ was spread on plates containing Müeller-Hinton agar (Himedia). Holes of approximately 5 × 3 mm were made in the agar and filled with 30 *μ*L of the fraction stock solutions (50 mg mL^−1^) and with 10 *μ*g mL^−1^ for compounds **1**, **2**, and **3**. After incubation at 37°C for 24 h, inhibition zones were measured in millimeters and compared to the controls. The antibiotic ciclopirox olamine (Uci-Farma) was used as the positive control due to its antibacterial properties [10]. Dimethylsulfoxide (DMSO) was used as a negative control. Tests were performed twice in triplicate. The minimum inhibitory concentration (MIC) of compound **1** was determined by applying a broth microdilution method followed by incubation at 37°C for 24 h and by observing media turbidity [11]. Tests were performed twice in triplicate.

### 2.6. Production of Herbal Soap

The active extract of *S. auriculata *(250 mg) was incorporated into a soap produced according to its patent 1005633-5 [12]. Later, the semi solid mixture was poured into a mold and allowed to solidify. Soap without the extract was also produced to be used as a reference product.

### 2.7. Antibacterial Assay of the Herbal Soap

The agar-dilution method was employed in an *in vitro *evaluation. The herbal soap (1.0 g) was dissolved in distilled water (50 mL) to obtain a 2% suspension. The suspension was vigorously shaken to dissolve the soap, to disperse the foam, and to homogenize the suspension. Next, 1.0 mL of the soap solution was added to 20 mL of sterile molten culture media in Petri dishes and allowed to set. One-hundred *μ*L of suspension containing 10^6^ CFU mL^−1^ of a resistant 4127 *S. aureus *strain was then streaked on the plates. After incubation at 37°C for 24 h, inhibition zones were compared to the control to observe the presence or absence of microbial growth.

## 3. Results and Discussion

**Figure 1:**
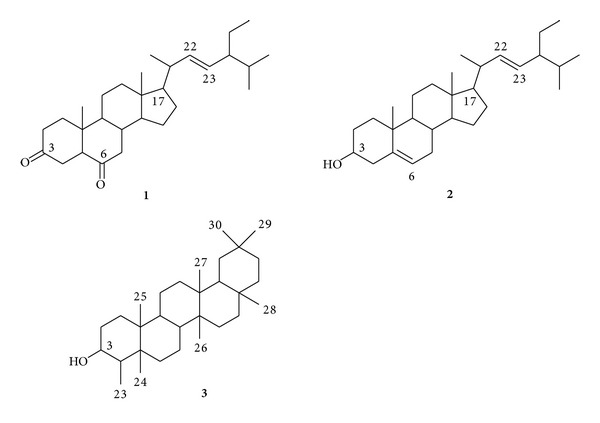
Isolated compounds from the active fraction of *Salvinia auriculata* roots.

Purification of the active fraction obtained from the bioassay-guided active extract of *S. auriculata *roots led to the isolation of two steroids and one triterpene. These isolated compounds were characterized as stigmasterone (**1**), stigmasterol (**2**), and friedelinol (**3**) (Figure 1).

Compound **1** was obtained as colorless needless, m.p. 187–189°C. The molecular formula of C_29_H_46_O_2_ was determined by HR-EI-MS [M+H]^+^ *m/z* = 426.6740. The fragment at *m*/*z* = 245 suggests the presence of two carbonyl groups [13], while the IV spectrum confirms the presence of carbonyl groups with absorption at 1715 cm^−1^. Careful analysis of ^1^H nuclear magnetic resonance (NMR) spectrum allowed for the observation of two double doublets at *δ* 5.20 (dd, *J* = 15.1, 8.4 Hz, H-22) and 5.08 (dd, *J* = 15.1, 8.4 Hz, H-23), which correspond to typical olefinic protons of steroid side chains. The nature of the double bond was assumed to be *trans *based on the value of the coupling constant *J* = 15.10 Hz from H-22/H-23. In the COSY spectrum, H-2*α* and H-2*β* showed cross peaks at *δ* 1.62 and *δ* 2.1 with 2H-1, whereas H-4*α* and H-4*β* presented cross correlations with H-5 at *δ* 2.6. Both protons at C-7 presented the same cross peak at *δ* 1.86 (H-8). In the ^13^C nuclear magnetic resonance (NMR) spectrum, two signals at *δ* 138.1 and 129.8 could be observed, confirming the presence of olefinic carbons C-22 and C-23, respectively. In addition, signals could be observed at *δ* 211.7 and *δ* 209.5, which represent the characteristics of two quaternary carbons of carbonyl groups, as defined by the Distortionless Enhancement by Polarization Transfer (DEPT) spectrum. According to the present study's data and compared to reports found in the literature [13, 14], compound **1** is in fact the stigmast-22-ene-3,6-dione, MP: 187–189°C; IR (KBr): 2925, 2853, 1715 cm^−1^; ^1^H NMR (300 MHz, CDCl_3_): *δ* 0.74 (3H, s, 18-CH_3_), 0.82 (3H, s, 26-CH_3_), 0.84 (3H, s, 29-CH_3_), 0.89 (3H, s, 27-CH_3_), 0.99 (3H, s, 19-CH_3_), 1.07 (3H, s, 21-CH_3_), 1.20 (1H, m, 28-H), 1.46 (1H, m, 28-H), 1.56 (1H, m, 24-H), 1.60 (1H, m, 25-H), 2.08 (1H, m, 20-H), 5.08 (1H, dd, *J* = 15.12 8.4 Hz, 23-H), 5.20 (1H, dd, *J* = 15.10, 8.4 Hz, 22-H); ^13^C NMR (75 MHz CDCl_3_): *δ* 12.20 (C-18), 12.56 (C-29), 18.93 (C-26), 21.40 (C-21), 21.89 (C-27), 26.20 (C-28), 32.64 (C-25), 40.68 (C-20), 51.47 (C-24), 129.80 (C-23), 138.10 (C-22), 209.50 (C-3), 211.70 (C-6); MS (EI, 70 eV): *m*/*z*(%) = 397 (7); 303 (3); 315 (13); 259 (3); 245 (10); 217 (5); 190 (7); 175 (3); 147 (8); 137 (6); 119 (83); HRMS-FAB:  *m*/*z*[M + H^+^] calcd for C_29_H_46_O_2_ 426.6743; found 426.6740. Compounds **2** and **3** are also known compounds and were identified by comparing the spectroscopic data with the reported values. All compounds were isolated from *S. auriculata* for the first time.

Compound **1** can only be detected in the final stages of plant development and is considered a rare plant constituent [15]. Steroids containing 3,6-dione groups within their structures present prominent biological activities, including anti-inflammatory, antiallergic, and allelopathy [16, 17]. Results concerning the antimicrobial activity of compound **1** are in accordance with findings from the literature regarding antimicrobial activity for this compound.

In a prior study [7], the hexane extract from *S. auriculata *roots presented a high activity against *S. aureus *strains isolated from animals with mastitis infection, as shown in Table 1.

Results from Table 1 are in accordance with those displayed for compound **1** (see Table 2), which exhibited inhibition zones as large as the hexane extract and even greater than the positive control, confirming that this compound may well be responsible for the activity of the extract. Compounds **2** and **3** showed no activity against tested bacteria.

Table 3 shows the MIC value for compound **1** and the biofilm inhibitory concentration (BIC) value for the active extract, as previously published [7].

The MIC values obtained for the active extract are lower than some values previously found in extracts with antimicrobial activity [18, 19]. Based on the Aligiannis criteria within MIC values (extracts can present strong (0.05 to 0.5 mg mL^−1^), moderate (0.6 to 1.5 mg mL^−1^), or weak activity (>1.5 mg mL^−1^)), compound **1**, as well as the active extract of *S. auriculata *with MIC values of 0.01 and 0.3 mg mL^−1^, respectively, can be considered strong inhibitors [20]. The values determined for BIC were similar to those found for antibiotic substances reported in the literature [21, 22].

By contrast, the herbal soap produced using the active extract of *S. auriculata* showed a high antimicrobial activity against the tested *S. aureus*. According to the results observed in the *in vitro* evaluation, no microbial growth could be observed on the plate containing the herbal soap of *S. auriculata* in relation to the control (ciclopirox olamine). This confirms that this extract does in fact have antimicrobial activity and could be used as an excipient for soap antiseptics.

## 4. Conclusion

In summary, the strong inhibitory effects of the active extract of *S. auriculata *against *S. aureus *strains can be attributed to compound **1**, a 3,6-dioxygenated steroid, found primarily in aquatic plants [10]. Moreover, this activity may well be associated with a synergism with other compounds that have not yet been isolated from the active extract. Nevertheless, the herbal soap produced with *S. auriculata *demonstrated a high inhibition against an *S. aureus *infection of cows' udders, indicating the potential of the plant as an excipient in the production of antiseptic soap aimed at cleansing the animals' udders before milking, in turn controlling bovine mastitis infections, especially on small farms. Therefore, it can be concluded that these findings are of high economic, industrial, and veterinary significance.

## Figures and Tables

**Table 1:** Antibacterial activity of hexane extract from *Salvinia auriculata *roots.

*S. aureus *	Hexane extract	Ciclopirox olamine	DMSO
Inhibition zones (mm ± SD)			
2221	20.67 ± 0.33	21.67 ± 0.67	0.00
3008	20.67 ± 0.88	21.33 ± 0.88	0.00
4072	20.00 ± 0.58	23.00 ± 0.58	0.00
3007	21.00 ± 0.33	21.00 ± 0.58	0.00
4163	20.00 ± 0.58	21.00 ± 0.58	0.00
4347	23.00 ± 0.58	21.00 ± 0.58	0.00

**Table 2:** Antibacterial activity of compounds isolated from *Salvinia auriculata *roots.

*S. aureus *	Compound **1**	Compound **2**	Compound **3**	Ciclopirox olamine	DMSO
Inhibition zones (mm ± SD)					
582	23.0 ± 0.58	0.00	0.00	17.0 ± 0.58	0.00
680	23.0 ± 0.60	0.00	0.00	18.0 ± 0.60	0.00
2221	21.0 ± 0.33	0.00	0.00	17.0 ± 0.58	0.00
4006	20.0 ± 0.67	0.00	0.00	17.0 ± 0.58	0.00
4127	20.0 ± 0.58	0.00	0.00	17.0 ± 0.50	0.00

**Table 3:** MIC and BIC values obtained from compound **1** and the active extract, respectively, on *Staphylococcus aureus*.

	MIC (mg mL^−1^)	BIC (mg mL^−1^)
Active extract	0.3	0.075
Compound **1**	0.01	—
Ciclopirox olamine	0.05	0.025

## References

[1] Meléndez PA, Capriles VA (2006). Antibacterial properties of tropical plants from Puerto Rico. *Phytomedicine*.

[2] Hu J-F, Garo E, Goering MG (2006). Bacterial biofilm inhibitors from *Diospyros dendo*. *Journal of Natural Products*.

[3] Vattem DA, Mihalik K, Crixell SH, McLean RJC (2007). Dietary phytochemicals as quorum sensing inhibitors. *Fitoterapia*.

[4] Özbay H, Alim A (2009). Antimicrobial activity of some water plants from the northeastern Anatolian region of Turkey. *Molecules*.

[5] Lorenzi H (2000). *Plantas Daninhas do Brasil: Terrestres, Aquáticas Parasitas e Tóxicas*.

[6] Soares DCF, de Oliveira EF, de Fátima Silva GD, Duarte LP, Pott VJ, Filho SAV (2008). *Salvinia auriculata*: aquatic bioindicator studied by instrumental neutron activation analysis (INAA). *Applied Radiation and Isotopes*.

[7] Rossi CC, Aguilar AP, Diaz MAN, Ribon ADOB (2011). Aquatic plants as potential sources of antimicrobial compounds active against bovine mastitis pathogens. *African Journal of Biotechnology*.

[8] LeBlanc SJ, Lissemore KD, Kelton DF, Duffield TF, Leslie KE (2006). Major advances in disease prevention in dairy cattle. *Journal of Dairy Science*.

[9] Secretaria de Estado e Agricultura Pecuária e Abastecimento de Minas Gerais. http://www.agricultura.mg.gov.br/.

[10] Jue SG, Dawson GW, Brogden RN (1985). Ciclopirox olamine 1% cream; a preliminary review of its antimicrobial activity and therapeutic use. *Drugs*.

[11] Caetano N, Saraiva A, Pereira R, Carvalho D, Pimentel MCB, Maia MBS (2002). Determinação da atividade antimicrobiana de extratos de plantas de uso popular como anti-inflamatório. *Brazilian Journal of Pharmacognosy*.

[12] Diaz MAN Composições domissaneantes à base de óleo de macaúba e extratos de *Salvinia auriculata* e seus derivados com ação terapêutica e seu uso para prevenção e/ou controle de mastite bovina.

[13] Georges P, Sylvestre M, Ruegger H, Bourgeois P (2006). Ketosteroids and hydroxyketosteroids, minor metabolites of sugarcane wax. *Steroids*.

[14] Isabel Fernández M, Pedro JR, Seoane E (1983). Constituents of a hexane extract of *Phoenix dactylifera*. *Phytochemistry*.

[15] Radulović NS, Dordević ND (2011). Steroids from poison hemlock (*Conium maculatum* L.): a GC-MS analysis. *Journal of the Serbian Chemical Society*.

[16] Aliotta G, Monaco P, Pinto G, Pollio A, Previtera L (1991). Potential allelochemicals from *Pistia stratiotes* L. *Journal of Chemical Ecology*.

[17] Tsai P-L, Wang J-P, Chang C-W, Kuo S-C, Lee Chao P-D (1998). Constituents and bioactive principles of *Polygonum chinensis*. *Phytochemistry*.

[18] Duarte MCT, Figueira GM, Pereira B, Magalhães PM, Delarmelina C (2004). Atividade antimicrobiana de extratos hidroalcoólicos de espécies da coleção de plantas medicinais. *Brazilian Journal of Pharmacognosy*.

[19] Virtuoso S, Davet A, Dias JFG (2005). Estudo preliminar da atividade antibacteriana das cascas de *Erythrina velutina* Willd., Fabaceae (Leguminosae). *Brazilian Journal of Pharmacognosy*.

[20] Aligiannis N, Kalpoutzakis E, Mitaku S, Chinou IB (2001). Composition and antimicrobial activity of the essential oils of two Origanum species. *Journal of Agricultural and Food Chemistry*.

[21] García-Castillo M, Morosini MI, Valverde A (2007). Differences in biofilm development and antibiotic susceptibility among *Streptococcus pneumoniae* isolates from cystic fibrosis samples and blood cultures. *Journal of Antimicrobial Chemotherapy*.

[22] Nostro A, Roccaro AS, Bisignano G (2007). Effects of oregano, carvacrol and thymol on *Staphylococcus aureus* and *Staphylococcus epidermidis* biofilms. *Journal of Medical Microbiology*.

